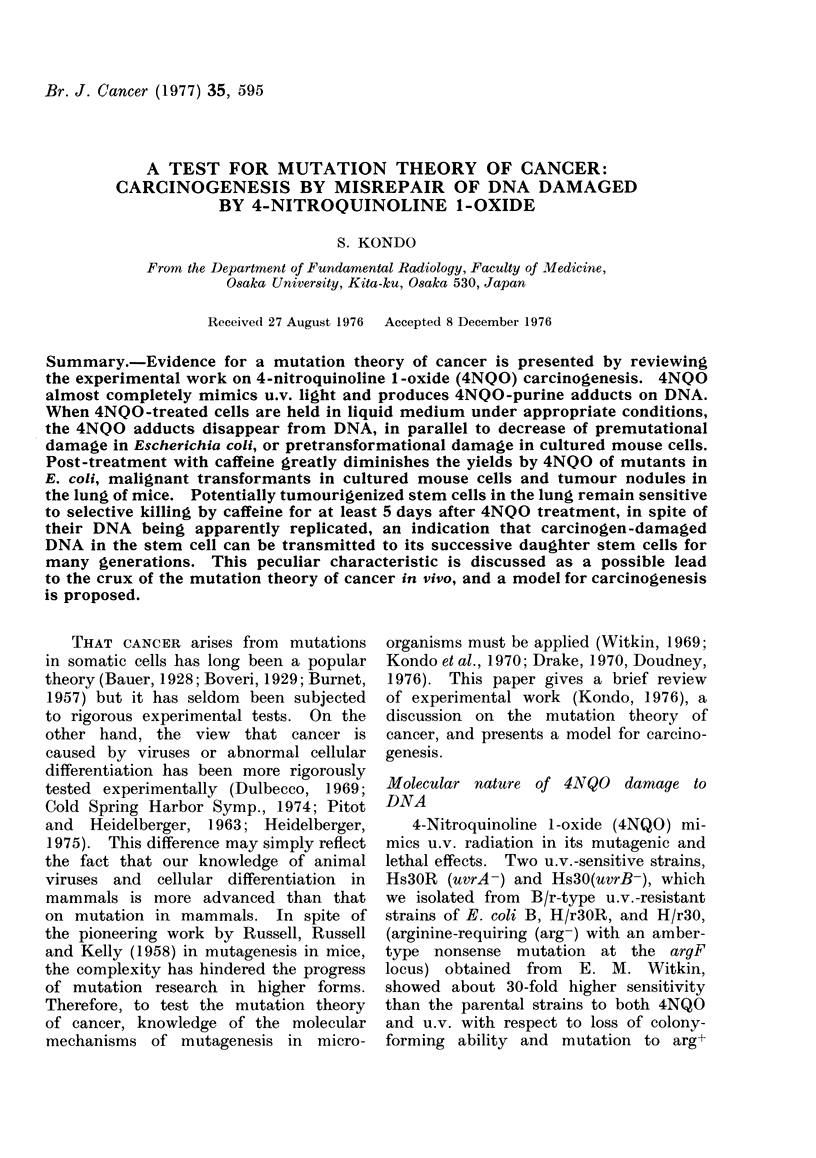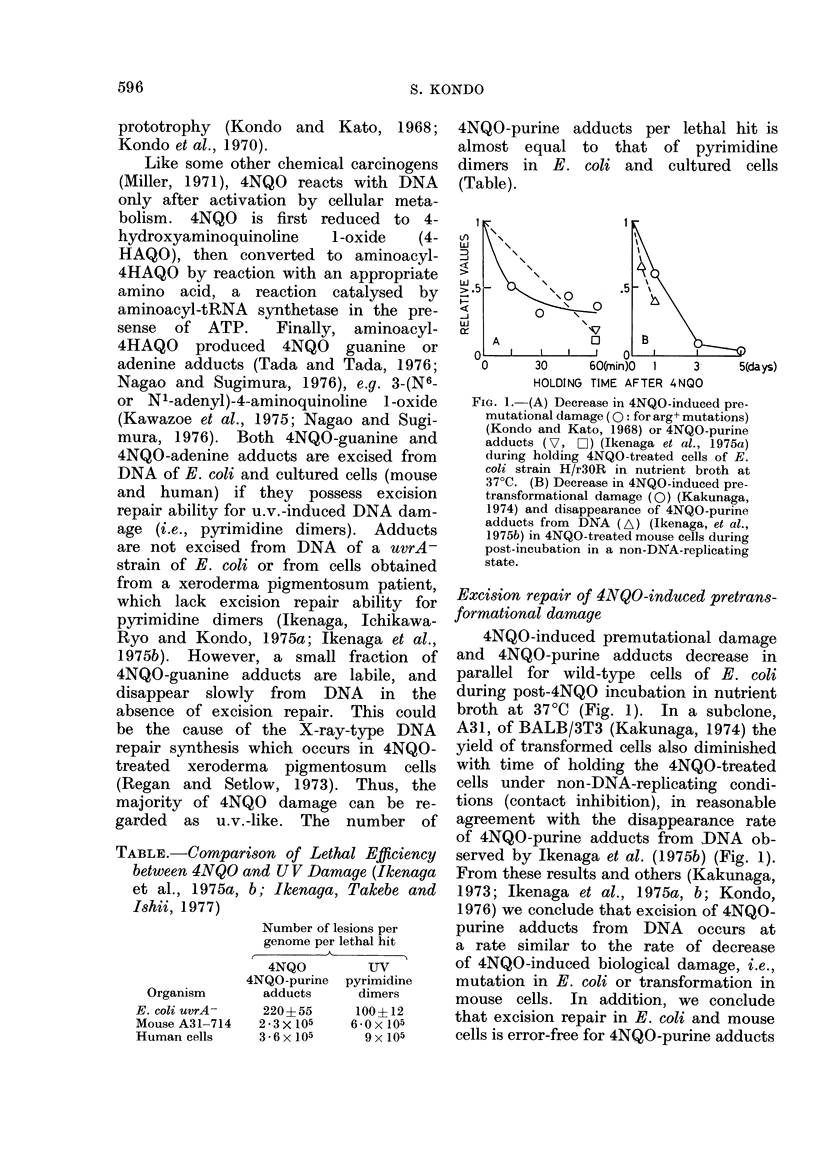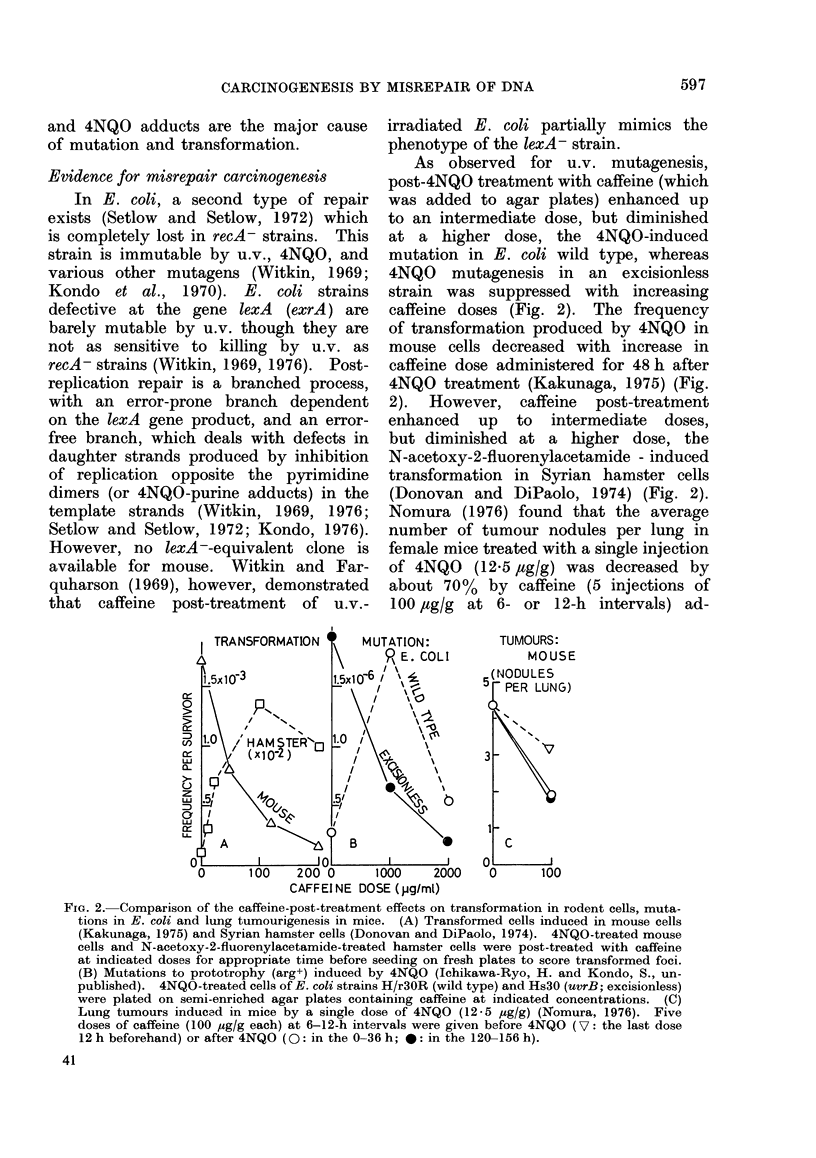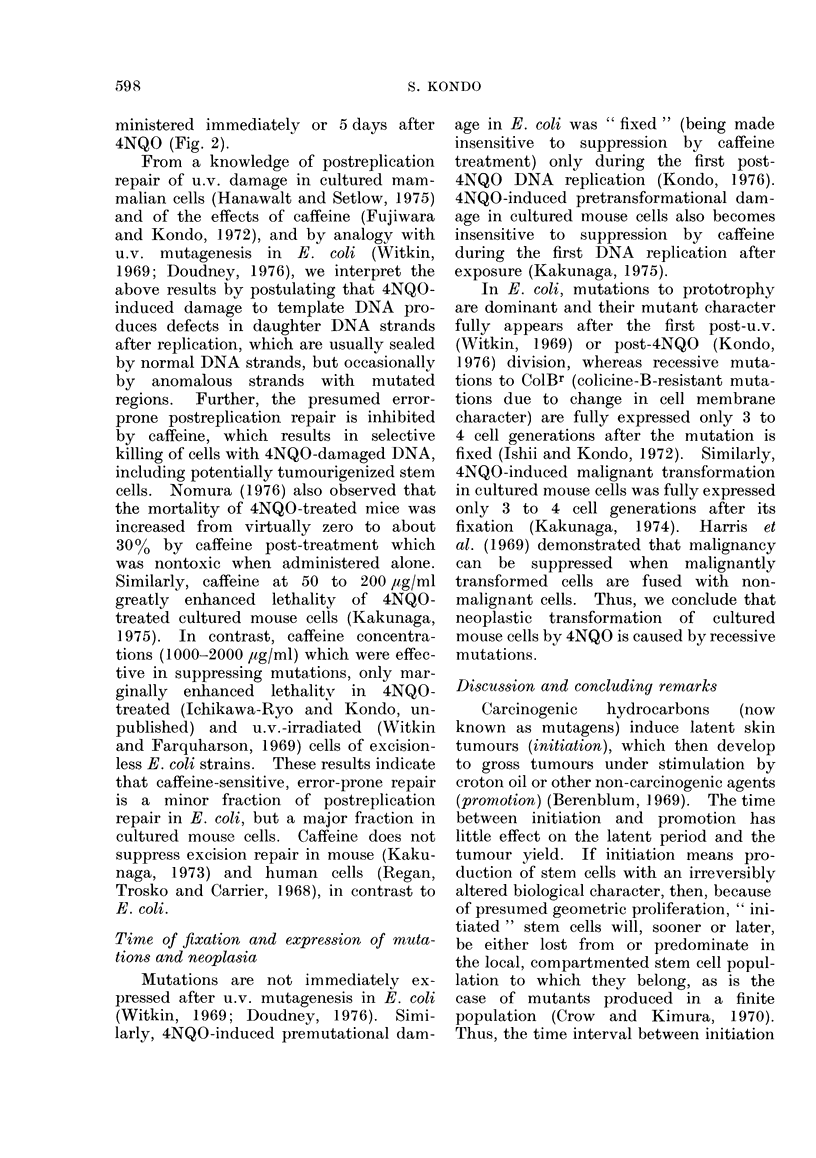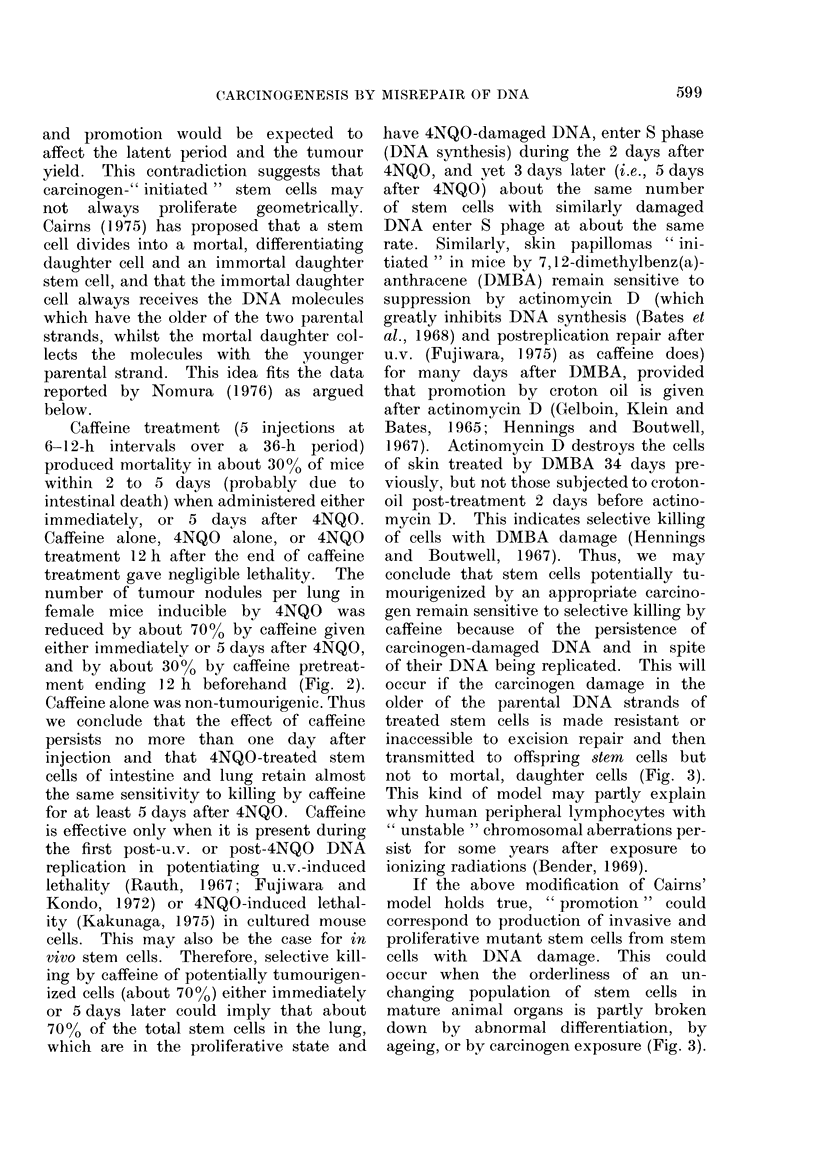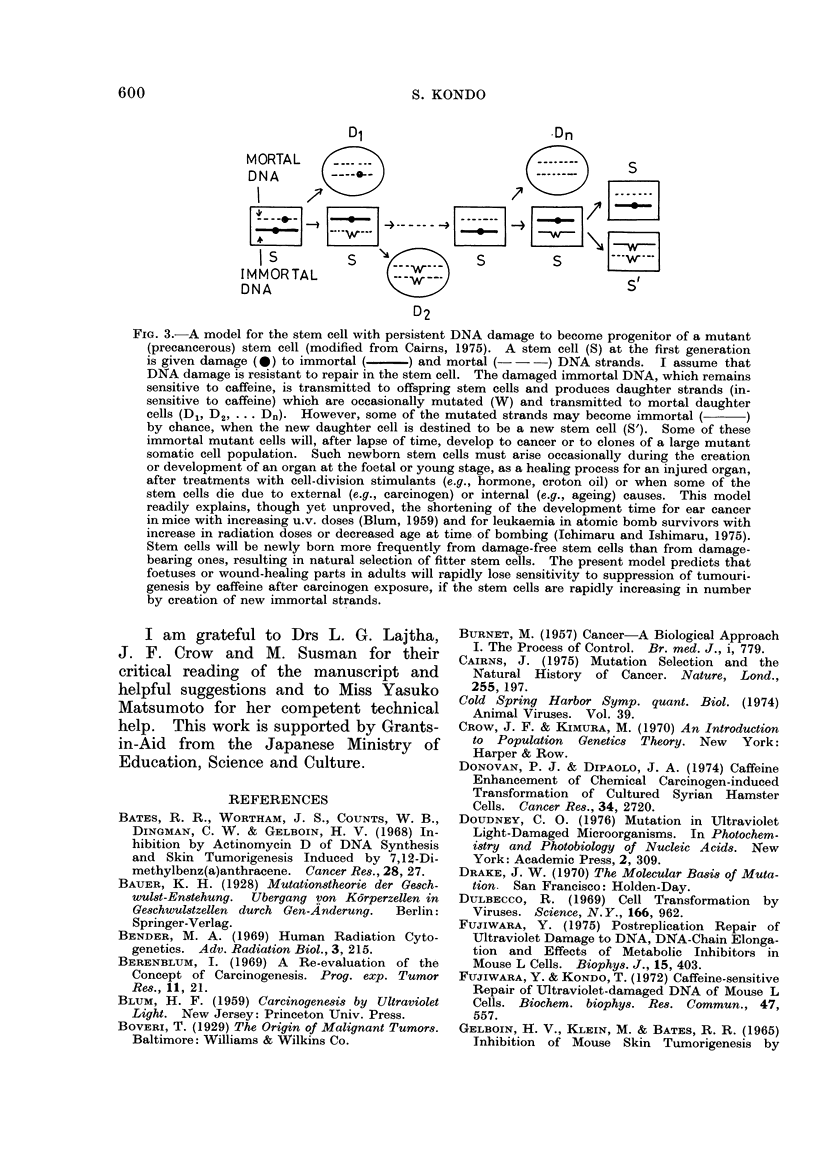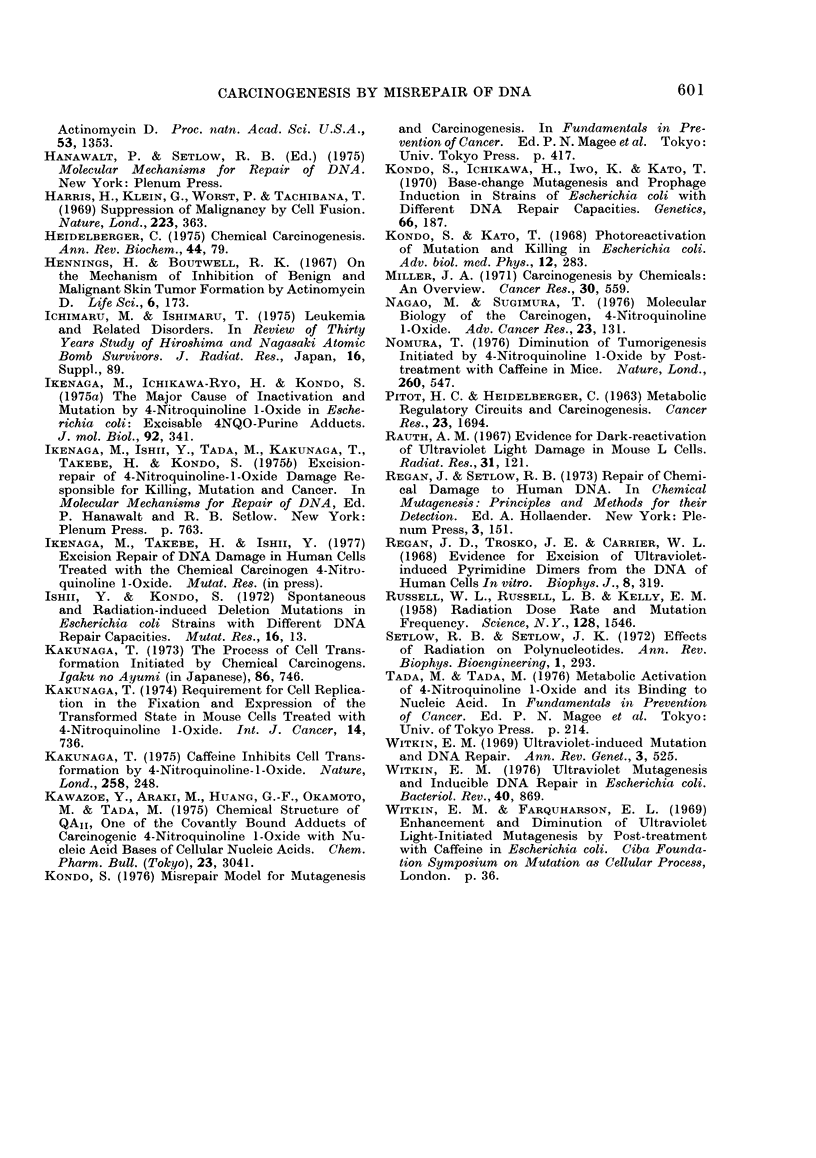# A test for mutation theory of cancer: carcinogenesis by misrepair of DNA damaged by 4-nitroquinoline 1-oxide.

**DOI:** 10.1038/bjc.1977.93

**Published:** 1977-05

**Authors:** S. Kondo

## Abstract

Evidence for a mutation theory of cancer is presented by reviewing the experimental work on 4-nitroquinoline 1-oxide (4NQO) carcinogenesis. 4NQO almost completely mimics u.v. light and produces 4NQO-purine adducts on DNA. When 4NQO-treated cells are held in liquid medium under appropriate conditions, the 4NQO adducts disappear from DNA, in parallel to decrease of premutational damage in Escherichia coli, or pretransformational damage in cultured mouse cells. Post-treatment with caffeine greatly diminishes the yields by 4NQO of mutants in E. coli, malignant transformants in cultured mouse cells and tumour nodules in the lung of mice. Potentially tumourigenized stem cells in the lung remain sensitive to selective killing by caffeine for at least 5 days after 4NQO treatment, in spite of their DNA being apparently replicated, an indication that carcinogen-damaged DNA in the stem cell can be transmitted to its successive daughter stem cells for many generations. This peculiar characteristic is discussed as a possible lead to the crux of the mutation theory of cancer in vivo, and a model for carcinogenesis is proposed.


*Br. J. Cancer* (1977) **35**, 595

### A TEST FOR MUTATION THEORY OF CANCER: CARCINOGENESIS BY MISREPAIR OF DNA DAMAGED BY 4-NITROQUINOLINE 1-OXIDE

S. KONDO

*From the Department of Fundamental Radiology, Faculty of Medicine, Osaka University, Kita-ku, Osaka* 530, *Japan*

Received 27 August 1976 Accepted 8 December 1976

**Summary.—Evidence for a mutation theory of cancer is presented by reviewing the experimental work on 4-nitroquinoline 1-oxide (4NQO) carcinogenesis. 4NQO almost completely mimics u.v. light and produces 4NQO-purine adducts on DNA. When 4NQO-treated cells are held in liquid medium under appropriate conditions, the 4NQO adducts disappear from DNA, in parallel to decrease of premutational damage in *Escherichia coli*, or pretransformational damage in cultured mouse cells. Post-treatment with caffeine greatly diminishes the yields by 4NQO of mutants in *E. coli*, malignant transformants in cultured mouse cells and tumour nodules in the lung of mice. Potentially tumourigenized stem cells in the lung remain sensitive to selective killing by caffeine for at least 5 days after 4NQO treatment, in spite of their DNA being apparently replicated, an indication that carcinogen-damaged DNA in the stem cell can be transmitted to its successive daughter stem cells for many generations. This peculiar characteristic is discussed as a possible lead to the crux of the mutation theory of cancer *in vivo*, and a model for carcinogenesis is proposed.**

THAT CANCER arises from mutations in somatic cells has long been a popular theory (Bauer, 1928; Boveri, 1929; Burnet, 1957) but it has seldom been subjected to rigorous experimental tests. On the other hand, the view that cancer is caused by viruses or abnormal cellular differentiation has been more rigorously tested experimentally (Dulbecco, 1969; Cold Spring Harbor Symp., 1974; Pitot and Heidelberger, 1963; Heidelberger, 1975). This difference may simply reflect the fact that our knowledge of animal viruses and cellular differentiation in mammals is more advanced than that on mutation in mammals. In spite of the pioneering work by Russell, Russell and Kelly (1958) in mutagenesis in mice, the complexity has hindered the progress of mutation research in higher forms. Therefore, to test the mutation theory of cancer, knowledge of the molecular mechanisms of mutagenesis in micro-organisms must be applied (Witkin, 1969; Kondo *et al.*, 1970; Drake, 1970, Doudney, 1976). This paper gives a brief review of experimental work (Kondo, 1976), a discussion on the mutation theory of cancer, and presents a model for carcinogenesis.

### *Molecular nature of 4NQO damage to DNA*

4-Nitroquinoline 1-oxide (4NQO) mimics u.v. radiation in its mutagenic and lethal effects. Two u.v.-sensitive strains, Hs30R (*uvrA*$^-$) and Hs30(*uvrB*$^-$), which we isolated from B/r-type u.v.-resistant strains of *E. coli* B, H/r30R, and H/r30, (arginine-requiring (arg$^-$) with an amber-type nonsense mutation at the *argF* locus) obtained from E. M. Witkin, showed about 30-fold higher sensitivity than the parental strains to both 4NQO and u.v. with respect to loss of colony-forming ability and mutation to arg$^+$

prototrophy (Kondo and Kato, 1968; Kondo *et al.*, 1970).

Like some other chemical carcinogens (Miller, 1971), 4NQO reacts with DNA only after activation by cellular metabolism. 4NQO is first reduced to 4-hydroxyaminoquinoline 1-oxide (4-HAQO), then converted to aminoacyl-4HAQO by reaction with an appropriate amino acid, a reaction catalysed by aminoacyl-tRNA synthetase in the presense of ATP. Finally, aminoacyl-4HAQO produced 4NQO guanine or adenine adducts (Tada and Tada, 1976; Nagao and Sugimura, 1976), *e.g.* 3-($N^6$- or $N^1$-adenyl)-4-aminoquinoline 1-oxide (Kawazoe *et al.*, 1975; Nagao and Sugimura, 1976). Both 4NQO-guanine and 4NQO-adenine adducts are excised from DNA of *E. coli* and cultured cells (mouse and human) if they possess excision repair ability for u.v.-induced DNA damage (*i.e.*, pyrimidine dimers). Adducts are not excised from DNA of a *uvrA*$^-$ strain of *E. coli* or from cells obtained from a xeroderma pigmentosum patient, which lack excision repair ability for pyrimidine dimers (Ikenaga, Ichikawa-Ryo and Kondo, 1975*a*; Ikenaga *et al.*, 1975*b*). However, a small fraction of 4NQO-guanine adducts are labile, and disappear slowly from DNA in the absence of excision repair. This could be the cause of the X-ray-type DNA repair synthesis which occurs in 4NQO-treated xeroderma pigmentosum cells (Regan and Setlow, 1973). Thus, the majority of 4NQO damage can be regarded as u.v.-like. The number of 4NQO-purine adducts per lethal hit is almost equal to that of pyrimidine dimers in *E. coli* and cultured cells (Table).

TABLE.—*Comparison of Lethal Efficiency between 4NQO and UV Damage (Ikenaga* et al., 1975*a*, *b; Ikenaga, Takebe and Ishii*, 1977)

| | Number of lesions per genome per lethal hit | |
|---|---|---|
| | 4NQO | UV |
| Organism | 4NQO-purine adducts | pyrimidine dimers |
| *E. coli uvrA*$^-$ | $220 \pm 55$ | $100 \pm 12$ |
| Mouse A31–714 | $2 \cdot 3 \times 10^5$ | $6 \cdot 0 \times 10^5$ |
| Human cells | $3 \cdot 6 \times 10^5$ | $9 \times 10^5$ |

FIG. 1.—(A) Decrease in 4NQO-induced premutational damage (○: for $arg^+$ mutations) (Kondo and Kato, 1968) or 4NQO-purine adducts (▽, □) (Ikenaga *et al.*, 1975*a*) during holding 4NQO-treated cells of *E. coli* strain H/r30R in nutrient broth at 37°C. (B) Decrease in 4NQO-induced pretransformational damage (○) (Kakunaga, 1974) and disappearance of 4NQO-purine adducts from DNA (△) (Ikenaga, *et al.*, 1975*b*) in 4NQO-treated mouse cells during post-incubation in a non-DNA-replicating state.

*Excision repair of 4NQO-induced pretransformational damage*

4NQO-induced premutational damage and 4NQO-purine adducts decrease in parallel for wild-type cells of *E. coli* during post-4NQO incubation in nutrient broth at 37°C (Fig. 1). In a subclone, A31, of BALB/3T3 (Kakunaga, 1974) the yield of transformed cells also diminished with time of holding the 4NQO-treated cells under non-DNA-replicating conditions (contact inhibition), in reasonable agreement with the disappearance rate of 4NQO-purine adducts from DNA observed by Ikenaga *et al.* (1975*b*) (Fig. 1). From these results and others (Kakunaga, 1973; Ikenaga *et al.*, 1975*a*, *b*; Kondo, 1976) we conclude that excision of 4NQO-purine adducts from DNA occurs at a rate similar to the rate of decrease of 4NQO-induced biological damage, *i.e.*, mutation in *E. coli* or transformation in mouse cells. In addition, we conclude that excision repair in *E. coli* and mouse cells is error-free for 4NQO-purine adducts and 4NQO adducts are the major cause of mutation and transformation.

*Evidence for misrepair carcinogenesis*

In *E. coli*, a second type of repair exists (Setlow and Setlow, 1972) which is completely lost in *recA*$^-$ strains. This strain is immutable by u.v., 4NQO, and various other mutagens (Witkin, 1969; Kondo *et al.*, 1970). *E. coli* strains defective at the gene *lexA* (*exrA*) are barely mutable by u.v. though they are not as sensitive to killing by u.v. as *recA*$^-$ strains (Witkin, 1969, 1976). Post-replication repair is a branched process, with an error-prone branch dependent on the *lexA* gene product, and an error-free branch, which deals with defects in daughter strands produced by inhibition of replication opposite the pyrimidine dimers (or 4NQO-purine adducts) in the template strands (Witkin, 1969, 1976; Setlow and Setlow, 1972; Kondo, 1976). However, no *lexA*$^-$-equivalent clone is available for mouse. Witkin and Farquharson (1969), however, demonstrated that caffeine post-treatment of u.v.-irradiated *E. coli* partially mimics the phenotype of the *lexA*$^-$ strain.

As observed for u.v. mutagenesis, post-4NQO treatment with caffeine (which was added to agar plates) enhanced up to an intermediate dose, but diminished at a higher dose, the 4NQO-induced mutation in *E. coli* wild type, whereas 4NQO mutagenesis in an excisionless strain was suppressed with increasing caffeine doses (Fig. 2). The frequency of transformation produced by 4NQO in mouse cells decreased with increase in caffeine dose administered for 48 h after 4NQO treatment (Kakunaga, 1975) (Fig. 2). However, caffeine post-treatment enhanced up to intermediate doses, but diminished at a higher dose, the N-acetoxy-2-fluorenylacetamide - induced transformation in Syrian hamster cells (Donovan and DiPaolo, 1974) (Fig. 2). Nomura (1976) found that the average number of tumour nodules per lung in female mice treated with a single injection of 4NQO (12·5 μg/g) was decreased by about 70% by caffeine (5 injections of 100 μg/g at 6- or 12-h intervals) ad-

FIG. 2.—Comparison of the caffeine-post-treatment effects on transformation in rodent cells, mutations in *E. coli* and lung tumourigenesis in mice. (A) Transformed cells induced in mouse cells (Kakunaga, 1975) and Syrian hamster cells (Donovan and DiPaolo, 1974). 4NQO-treated mouse cells and N-acetoxy-2-fluorenylacetamide-treated hamster cells were post-treated with caffeine at indicated doses for appropriate time before seeding on fresh plates to score transformed foci. (B) Mutations to prototrophy (arg$^+$) induced by 4NQO (Ichikawa-Ryo, H. and Kondo, S., unpublished). 4NQO-treated cells of *E. coli* strains H/r30R (wild type) and Hs30 (*uvrB*; excisionless) were plated on semi-enriched agar plates containing caffeine at indicated concentrations. (C) Lung tumours induced in mice by a single dose of 4NQO (12·5 μg/g) (Nomura, 1976). Five doses of caffeine (100 μg/g each) at 6–12-h intervals were given before 4NQO (▽: the last dose 12 h beforehand) or after 4NQO (○: in the 0–36 h; ●: in the 120–156 h).

ministered immediately or 5 days after 4NQO (Fig. 2).

From a knowledge of postreplication repair of u.v. damage in cultured mammalian cells (Hanawalt and Setlow, 1975) and of the effects of caffeine (Fujiwara and Kondo, 1972), and by analogy with u.v. mutagenesis in *E. coli* (Witkin, 1969; Doudney, 1976), we interpret the above results by postulating that 4NQO-induced damage to template DNA produces defects in daughter DNA strands after replication, which are usually sealed by normal DNA strands, but occasionally by anomalous strands with mutated regions. Further, the presumed error-prone postreplication repair is inhibited by caffeine, which results in selective killing of cells with 4NQO-damaged DNA, including potentially tumourigenized stem cells. Nomura (1976) also observed that the mortality of 4NQO-treated mice was increased from virtually zero to about 30% by caffeine post-treatment which was nontoxic when administered alone. Similarly, caffeine at 50 to 200 $\mu$g/ml greatly enhanced lethality of 4NQO-treated cultured mouse cells (Kakunaga, 1975). In contrast, caffeine concentrations (1000–2000 $\mu$g/ml) which were effective in suppressing mutations, only marginally enhanced lethality in 4NQO-treated (Ichikawa-Ryo and Kondo, unpublished) and u.v.-irradiated (Witkin and Farquharson, 1969) cells of excisionless *E. coli* strains. These results indicate that caffeine-sensitive, error-prone repair is a minor fraction of postreplication repair in *E. coli*, but a major fraction in cultured mouse cells. Caffeine does not suppress excision repair in mouse (Kakunaga, 1973) and human cells (Regan, Trosko and Carrier, 1968), in contrast to *E. coli*.

*Time of fixation and expression of mutations and neoplasia*

Mutations are not immediately expressed after u.v. mutagenesis in *E. coli* (Witkin, 1969; Doudney, 1976). Similarly, 4NQO-induced premutational damage in *E. coli* was " fixed " (being made insensitive to suppression by caffeine treatment) only during the first post-4NQO DNA replication (Kondo, 1976). 4NQO-induced pretransformational damage in cultured mouse cells also becomes insensitive to suppression by caffeine during the first DNA replication after exposure (Kakunaga, 1975).

In *E. coli*, mutations to prototrophy are dominant and their mutant character fully appears after the first post-u.v. (Witkin, 1969) or post-4NQO (Kondo, 1976) division, whereas recessive mutations to $ColB^r$ (colicine-B-resistant mutations due to change in cell membrane character) are fully expressed only 3 to 4 cell generations after the mutation is fixed (Ishii and Kondo, 1972). Similarly, 4NQO-induced malignant transformation in cultured mouse cells was fully expressed only 3 to 4 cell generations after its fixation (Kakunaga, 1974). Harris *et al.* (1969) demonstrated that malignancy can be suppressed when malignantly transformed cells are fused with non-malignant cells. Thus, we conclude that neoplastic transformation of cultured mouse cells by 4NQO is caused by recessive mutations.

*Discussion and concluding remarks*

Carcinogenic hydrocarbons (now known as mutagens) induce latent skin tumours (*initiation*), which then develop to gross tumours under stimulation by croton oil or other non-carcinogenic agents (*promotion*) (Berenblum, 1969). The time between initiation and promotion has little effect on the latent period and the tumour yield. If initiation means production of stem cells with an irreversibly altered biological character, then, because of presumed geometric proliferation, " initiated " stem cells will, sooner or later, be either lost from or predominate in the local, compartmented stem cell population to which they belong, as is the case of mutants produced in a finite population (Crow and Kimura, 1970). Thus, the time interval between initiation and promotion would be expected to affect the latent period and the tumour yield. This contradiction suggests that carcinogen-" initiated " stem cells may not always proliferate geometrically. Cairns (1975) has proposed that a stem cell divides into a mortal, differentiating daughter cell and an immortal daughter stem cell, and that the immortal daughter cell always receives the DNA molecules which have the older of the two parental strands, whilst the mortal daughter collects the molecules with the younger parental strand. This idea fits the data reported by Nomura (1976) as argued below.

Caffeine treatment (5 injections at 6–12-h intervals over a 36-h period) produced mortality in about 30% of mice within 2 to 5 days (probably due to intestinal death) when administered either immediately, or 5 days after 4NQO. Caffeine alone, 4NQO alone, or 4NQO treatment 12 h after the end of caffeine treatment gave negligible lethality. The number of tumour nodules per lung in female mice inducible by 4NQO was reduced by about 70% by caffeine given either immediately or 5 days after 4NQO, and by about 30% by caffeine pretreatment ending 12 h beforehand (Fig. 2). Caffeine alone was non-tumourigenic. Thus we conclude that the effect of caffeine persists no more than one day after injection and that 4NQO-treated stem cells of intestine and lung retain almost the same sensitivity to killing by caffeine for at least 5 days after 4NQO. Caffeine is effective only when it is present during the first post-u.v. or post-4NQO DNA replication in potentiating u.v.-induced lethality (Rauth, 1967; Fujiwara and Kondo, 1972) or 4NQO-induced lethality (Kakunaga, 1975) in cultured mouse cells. This may also be the case for *in vivo* stem cells. Therefore, selective killing by caffeine of potentially tumourigenized cells (about 70%) either immediately or 5 days later could imply that about 70% of the total stem cells in the lung, which are in the proliferative state and have 4NQO-damaged DNA, enter S phase (DNA synthesis) during the 2 days after 4NQO, and yet 3 days later (*i.e.*, 5 days after 4NQO) about the same number of stem cells with similarly damaged DNA enter S phage at about the same rate. Similarly, skin papillomas " initiated " in mice by 7,12-dimethylbenz(a)-anthracene (DMBA) remain sensitive to suppression by actinomycin D (which greatly inhibits DNA synthesis (Bates *et al.*, 1968) and postreplication repair after u.v. (Fujiwara, 1975) as caffeine does) for many days after DMBA, provided that promotion by croton oil is given after actinomycin D (Gelboin, Klein and Bates, 1965; Hennings and Boutwell, 1967). Actinomycin D destroys the cells of skin treated by DMBA 34 days previously, but not those subjected to croton-oil post-treatment 2 days before actinomycin D. This indicates selective killing of cells with DMBA damage (Hennings and Boutwell, 1967). Thus, we may conclude that stem cells potentially tumourigenized by an appropriate carcinogen remain sensitive to selective killing by caffeine because of the persistence of carcinogen-damaged DNA and in spite of their DNA being replicated. This will occur if the carcinogen damage in the older of the parental DNA strands of treated stem cells is made resistant or inaccessible to excision repair and then transmitted to offspring *stem* cells but not to mortal, daughter cells (Fig. 3). This kind of model may partly explain why human peripheral lymphocytes with " unstable " chromosomal aberrations persist for some years after exposure to ionizing radiations (Bender, 1969).

If the above modification of Cairns' model holds true, " promotion " could correspond to production of invasive and proliferative mutant stem cells from stem cells with DNA damage. This could occur when the orderliness of an unchanging population of stem cells in mature animal organs is partly broken down by abnormal differentiation, by ageing, or by carcinogen exposure (Fig. 3).

FIG. 3.—A model for the stem cell with persistent DNA damage to become progenitor of a mutant (precancerous) stem cell (modified from Cairns, 1975). A stem cell (S) at the first generation is given damage (●) to immortal (———) and mortal (— — — —) DNA strands. I assume that DNA damage is resistant to repair in the stem cell. The damaged immortal DNA, which remains sensitive to caffeine, is transmitted to offspring stem cells and produces daughter strands (insensitive to caffeine) which are occasionally mutated (W) and transmitted to mortal daughter cells ($D_1$, $D_2$, . . . $D_n$). However, some of the mutated strands may become immortal (———) by chance, when the new daughter cell is destined to be a new stem cell (S′). Some of these immortal mutant cells will, after lapse of time, develop to cancer or to clones of a large mutant somatic cell population. Such newborn stem cells must arise occasionally during the creation or development of an organ at the foetal or young stage, as a healing process for an injured organ, after treatments with cell-division stimulants (*e.g.*, hormone, croton oil) or when some of the stem cells die due to external (*e.g.*, carcinogen) or internal (*e.g.*, ageing) causes. This model readily explains, though yet unproved, the shortening of the development time for ear cancer in mice with increasing u.v. doses (Blum, 1959) and for leukaemia in atomic bomb survivors with increase in radiation doses or decreased age at time of bombing (Ichimaru and Ishimaru, 1975). Stem cells will be newly born more frequently from damage-free stem cells than from damage-bearing ones, resulting in natural selection of fitter stem cells. The present model predicts that foetuses or wound-healing parts in adults will rapidly lose sensitivity to suppression of tumourigenesis by caffeine after carcinogen exposure, if the stem cells are rapidly increasing in number by creation of new immortal strands.

I am grateful to Drs L. G. Lajtha, J. F. Crow and M. Susman for their critical reading of the manuscript and helpful suggestions and to Miss Yasuko Matsumoto for her competent technical help. This work is supported by Grants-in-Aid from the Japanese Ministry of Education, Science and Culture.

### REFERENCES

BATES, R. R., WORTHAM, J. S., COUNTS, W. B., DINGMAN, C. W. & GELBOIN, H. V. (1968) Inhibition by Actinomycin D of DNA Synthesis and Skin Tumorigenesis Induced by 7,12-Dimethylbenz(a)anthracene. *Cancer Res.*, **28**, 27.

BAUER, K. H. (1928) *Mutationstheorie der Geschwulst-Enstehung. Ubergang von Körperzellen in Geschwulstzellen durch Gen-Änderung.* Berlin: Springer-Verlag.

BENDER, M. A. (1969) Human Radiation Cytogenetics. *Adv. Radiation Biol.*, **3**, 215.

BERENBLUM, I. (1969) A Re-evaluation of the Concept of Carcinogenesis. *Prog. exp. Tumor Res.*, **11**, 21.

BLUM, H. F. (1959) *Carcinogenesis by Ultraviolet Light.* New Jersey: Princeton Univ. Press.

BOVERI, T. (1929) *The Origin of Malignant Tumors.* Baltimore: Williams & Wilkins Co.

BURNET, M. (1957) Cancer—A Biological Approach I. The Process of Control. *Br. med. J.*, i, 779.

CAIRNS, J. (1975) Mutation Selection and the Natural History of Cancer. *Nature, Lond.*, **255**, 197.

*Cold Spring Harbor Symp. quant. Biol.* (1974) Animal Viruses. Vol. 39.

CROW, J. F. & KIMURA, M. (1970) *An Introduction to Population Genetics Theory.* New York: Harper & Row.

DONOVAN, P. J. & DIPAOLO, J. A. (1974) Caffeine Enhancement of Chemical Carcinogen-induced Transformation of Cultured Syrian Hamster Cells. *Cancer Res.*, **34**, 2720.

DOUDNEY, C. O. (1976) Mutation in Ultraviolet Light-Damaged Microorganisms. In *Photochemistry and Photobiology of Nucleic Acids.* New York: Academic Press, **2**, 309.

DRAKE, J. W. (1970) *The Molecular Basis of Mutation.* San Francisco: Holden-Day.

DULBECCO, R. (1969) Cell Transformation by Viruses. *Science, N.Y.*, **166**, 962.

FUJIWARA, Y. (1975) Postreplication Repair of Ultraviolet Damage to DNA, DNA-Chain Elongation and Effects of Metabolic Inhibitors in Mouse L Cells. *Biophys. J.*, **15**, 403.

FUJIWARA, Y. & KONDO, T. (1972) Caffeine-sensitive Repair of Ultraviolet-damaged DNA of Mouse L Cells. *Biochem. biophys. Res. Commun.*, **47**, 557.

GELBOIN, H. V., KLEIN, M. & BATES, R. R. (1965) Inhibition of Mouse Skin Tumorigenesis by

Actinomycin D. *Proc. natn. Acad. Sci. U.S.A.*, **53**, 1353.

HANAWALT, P. & SETLOW, R. B. (Ed.) (1975) *Molecular Mechanisms for Repair of DNA*. New York: Plenum Press.

HARRIS, H., KLEIN, G., WORST, P. & TACHIBANA, T. (1969) Suppression of Malignancy by Cell Fusion. *Nature, Lond.*, **223**, 363.

HEIDELBERGER, C. (1975) Chemical Carcinogenesis. *Ann. Rev. Biochem.*, **44**, 79.

HENNINGS, H. & BOUTWELL, R. K. (1967) On the Mechanism of Inhibition of Benign and Malignant Skin Tumor Formation by Actinomycin D. *Life Sci.*, **6**, 173.

ICHIMARU, M. & ISHIMARU, T. (1975) Leukemia and Related Disorders. In *Review of Thirty Years Study of Hiroshima and Nagasaki Atomic Bomb Survivors. J. Radiat. Res.*, Japan, **16**, Suppl., 89.

IKENAGA, M., ICHIKAWA-RYO, H. & KONDO, S. (1975*a*) The Major Cause of Inactivation and Mutation by 4-Nitroquinoline 1-Oxide in *Escherichia coli*: Excisable 4NQO-Purine Adducts. *J. mol. Biol.*, **92**, 341.

IKENAGA, M., ISHII, Y., TADA, M., KAKUNAGA, T., TAKEBE, H. & KONDO, S. (1975*b*) Excision-repair of 4-Nitroquinoline-1-Oxide Damage Responsible for Killing, Mutation and Cancer. In *Molecular Mechanisms for Repair of DNA*, Ed. P. Hanawalt and R. B. Setlow. New York: Plenum Press. p. 763.

IKENAGA, M., TAKEBE, H. & ISHII, Y. (1977) Excision Repair of DNA Damage in Human Cells Treated with the Chemical Carcinogen 4-Nitroquinoline 1-Oxide. *Mutat. Res.* (in press).

ISHII, Y. & KONDO, S. (1972) Spontaneous and Radiation-induced Deletion Mutations in *Escherichia coli* Strains with Different DNA Repair Capacities. *Mutat. Res.*, **16**, 13.

KAKUNAGA, T. (1973) The Process of Cell Transformation Initiated by Chemical Carcinogens. *Igaku no Ayumi* (in Japanese), **86**, 746.

KAKUNAGA, T. (1974) Requirement for Cell Replication in the Fixation and Expression of the Transformed State in Mouse Cells Treated with 4-Nitroquinoline 1-Oxide. *Int. J. Cancer*, **14**, 736.

KAKUNAGA, T. (1975) Caffeine Inhibits Cell Transformation by 4-Nitroquinoline-1-Oxide. *Nature, Lond.*, **258**, 248.

KAWAZOE, Y., ARAKI, M., HUANG, G.-F., OKAMOTO, M. & TADA, M. (1975) Chemical Structure of $QA_{II}$, One of the Covantly Bound Adducts of Carcinogenic 4-Nitroquinoline 1-Oxide with Nucleic Acid Bases of Cellular Nucleic Acids. *Chem. Pharm. Bull.* (*Tokyo*), **23**, 3041.

KONDO, S. (1976) Misrepair Model for Mutagenesis and Carcinogenesis. In *Fundamentals in Prevention of Cancer*. Ed. P. N. Magee *et al.* Tokyo: Univ. Tokyo Press. p. 417.

KONDO, S., ICHIKAWA, H., IWO, K. & KATO, T. (1970) Base-change Mutagenesis and Prophage Induction in Strains of *Escherichia coli* with Different DNA Repair Capacities. *Genetics*, **66**, 187.

KONDO, S. & KATO, T. (1968) Photoreactivation of Mutation and Killing in *Escherichia coli*. *Adv. biol. med. Phys.*, **12**, 283.

MILLER, J. A. (1971) Carcinogenesis by Chemicals: An Overview. *Cancer Res.*, **30**, 559.

NAGAO, M. & SUGIMURA, T. (1976) Molecular Biology of the Carcinogen, 4-Nitroquinoline 1-Oxide. *Adv. Cancer Res.*, **23**, 131.

NOMURA, T. (1976) Diminution of Tumorigenesis Initiated by 4-Nitroquinoline 1-Oxide by Post-treatment with Caffeine in Mice. *Nature, Lond.*, **260**, 547.

PITOT, H. C. & HEIDELBERGER, C. (1963) Metabolic Regulatory Circuits and Carcinogenesis. *Cancer Res.*, **23**, 1694.

RAUTH, A. M. (1967) Evidence for Dark-reactivation of Ultraviolet Light Damage in Mouse L Cells. *Radiat. Res.*, **31**, 121.

REGAN, J. & SETLOW, R. B. (1973) Repair of Chemical Damage to Human DNA. In *Chemical Mutagenesis: Principles and Methods for their Detection*. Ed. A. Hollaender. New York: Plenum Press, **3**, 151.

REGAN, J. D., TROSKO, J. E. & CARRIER, W. L. (1968) Evidence for Excision of Ultraviolet-induced Pyrimidine Dimers from the DNA of Human Cells *In vitro*. *Biophys. J.*, **8**, 319.

RUSSELL, W. L., RUSSELL, L. B. & KELLY, E. M. (1958) Radiation Dose Rate and Mutation Frequency. *Science, N.Y.*, **128**, 1546.

SETLOW, R. B. & SETLOW, J. K. (1972) Effects of Radiation on Polynucleotides. *Ann. Rev. Biophys. Bioengineering*, **1**, 293.

TADA, M. & TADA, M. (1976) Metabolic Activation of 4-Nitroquinoline 1-Oxide and its Binding to Nucleic Acid. In *Fundamentals in Prevention of Cancer*. Ed. P. N. Magee *et al.* Tokyo: Univ. of Tokyo Press. p. 214.

WITKIN, E. M. (1969) Ultraviolet-induced Mutation and DNA Repair. *Ann. Rev. Genet.*, **3**, 525.

WITKIN, E. M. (1976) Ultraviolet Mutagenesis and Inducible DNA Repair in *Escherichia coli*. *Bacteriol. Rev.*, **40**, 869.

WITKIN, E. M. & FARQUHARSON, E. L. (1969) Enhancement and Diminution of Ultraviolet Light-Initiated Mutagenesis by Post-treatment with Caffeine in *Escherichia coli*. *Ciba Foundation Symposium on Mutation as Cellular Process*, London. p. 36.